# Supplementary material for: Impact of the COVID-19 pandemic and multiplex polymerase chain reaction test on outpatient antibiotic prescriptions for pediatric respiratory infection
Source: PLoS One. 2023 Jan 3;18(1):e0278932. doi: 10.1371/journal.pone.0278932 (PMC9810151; doi:10.1371/journal.pone.0278932)
Supplement: S1 Table — (DOCX) [file pone.0278932.s001.docx]

Supplementary Table S1. Patient characteristics and monthly antimicrobial prescription rates during the pre-pandemic and pandemic periods

|  | **<3 months** | | **3–11 months** | | **1–4 years** | | | **5–9 years** | | **10–14 years** | | **15–19 years** | | |
| --- | --- | --- | --- | --- | --- | --- | --- | --- | --- | --- | --- | --- | --- | --- |
|  | **Jan 2019–Mar 2020** | **Apr 2020–Dec 2021** | **Jan 2019–Mar 2020** | **Apr 2020–Dec 2021** | **Jan 2019–Mar 2020** | **Apr 2020–Dec 2021** | | **Jan 2019–Mar 2020** | **Apr 2020–Dec 2021** | **Jan 2019–Mar 2020** | **Apr 2020–Dec 2021** | **Jan 2019–Mar 2020** | **Apr 2020–Dec 2021** | |
| Average laboratory tests (min-max) | 6.0 (3–12) | 3.0 (0–11) | 12.9 (5–22) | 8.0 (1–26) | 52.7 (25–68) | 37.4 (16–94) | | 19.5 (8–38) | 8.7 (1–17) | 7.9 (1–18) | 5.4 (1–16) | 0.9 (0–3) | 1.0 (0–3) | |
| Sex % male (min-max) | 55.9 (25.0–83.3) | 63.3 (0.0–100) | 49.5 (25.0–71.4) | 53.7 (0.0–100) | 52.8 (41.3–63.2) | 56.3 (25.0–73.9) | | 57.9 (23.1–81.8) | 51.4 (0.0–100) | 45.5 (0.0–71.4) | 48.7 (0.0–100) | 59.3 (0.0–100) | 66.7 (0.0–100) | |
| Antibiotics prescribed % (min-max) |  |  |  |  |  |  | |  |  |  |  |  |  | |
| Overall antimicrobials | 10.8 (0.0–40.0) | 9.3 (0.0–33.3) | 33.1 (0.0–71.4) | 20.9 (0.0–100) | 41.8 (16.9–57.5) | 24.4 (12.3–41.9) | | 41.0 (15.4–64.7) | 17.0 (0.0–85.7) | 34.7 (0.0–66.7) | 17.3 (0.0–66.7) | 59.3 (0.0–100) | 26.9 (0.0–100) | |
| Penicillins | 2.3 (0.0–25.0) | 0.0 | 4.4 (0.0–28.6) | 0.0 | 4.1 (0.0–7.7) | 0.9 (0.0–5.6) | | 0.8 (0.0–9.1) | 1.7 (0.0–14.3) | 1.3 (0.0–14.3) | 0.6 (0.0–12.5) | 0.0 | 7.7 (0.0–100) | |
| Cephalosporins | 8.5 (0.0–40.0) | 9.3 (0.0–33.3) | 27.8 (0.0–60.0) | 19.0 (0.0–100) | 37.6 (15.4–57.5) | | 21.9 (6.3–41.9) | 34.9 (7.7–58.8) | 15.3 (0.0–71.4) | 28.9 (0.0–66.7) | 14.8 (0.0–50.0) | 48.1 (0.0–100) | 19.2 (0.0–100) |  |
| Quinolones | 0.0 | 0.0 | 0.0 | 0.0 | 0.0 | | 0.0 | 0.4 (0.0–6.7) | 0.0 | 0.0 | 0.0 | 0.0 | 0.0 |  |
| Macrolides | 0.0 | 0.5 (0.0–9.1) | 4.3 (0.0–22.2) | 1.4 (0.0–20.0) | 4.8 (0.0–12.5) | 3.1 (0.0–10.0) | | 7.5 (0.0–15.4) | 0.4 (0.0–9.1) | 4.4 (0.0–25.0) | 1.9 (0.0–33.3) | 11.1 (0.0–100) | 11.5 (0.0–100) | |
| Sulfamethoxazole-trimethoprim | 0.0 | 0.0 | 0.0 | 0.0 | 0.0 | 0.0 | | 0.0 | 0.0 | 0.5 (0.0–7.1) | 0.0 | 0.0 | 0.0 | |
| Other antibacterials | 0.0 | 0.0 | 0.4 (0.0–6.7) | 0.0 | 0.0 | 0.5 (0.0–4.3) | | 0.0 | 0.3 (0.0–5.9) | 1.3 (0.0–12.5) | 1.0 (0.0–12.5) | 0.0 | 0.0 | |
