# Supplementary material for: Impact of the COVID-19 pandemic and multiplex polymerase chain reaction test on outpatient antibiotic prescriptions for pediatric respiratory infection
Source: PLoS One. 2023 Jan 3;18(1):e0278932. doi: 10.1371/journal.pone.0278932 (PMC9810151; doi:10.1371/journal.pone.0278932)
Supplement: Attachment — Submitted filename: Editor_and_Reviewer_comments.docx [file pone.0278932.s002.docx]

Dear Editor and Reviewers:

We thank you for reviewing our manuscript and for providing constructive comments and suggestions. Our responses to the comments are provided below.

1. Compared with the pre pandemic period, age rate of patients less than 3 months is declining in pandemic period shown in Table 1. However, antibiotics prescribed rate of cephalosporins increases from 8.5 to 9.3 in Supplementary Table S1. Is this increase statistically significant? If so, why does this phenomenon occur in the youngest infants?

Author response 1: We thank you for your suggestion. The rate of cephalosporin antibiotic prescriptions for patients younger than 3 months was not significantly different between the pre-pandemic and pandemic periods with a p-value of 0.871. In addition, the use of antimicrobials for patients under 3 months differs from other age groups, even for simple fevers, and the risk of severe illness requiring antimicrobial treatment should be considered in this age group. We believe that it is difficult to evaluate the changes in antimicrobial use for this age group in the same way as for other age groups.

2. The RAT period has crossed pre- and pandemic period, however, the multiplex PCR period only covers pandemic period. Whether the data in the post-pandemic period still support the observation results in this paper is very promising.

Author response 2: We thank you for pointing this out. We will also analyze the Multiplex PCR data during the pandemic period as a future issue.

3. Only one P value (=0.33) has two decimal places reserved in Table 2, the others are all three. However, from abstract to text, most P values tend to be two digits on Line 37, 38, 180, 181, 193, 195, 197, and 200 etc., and three on Line 190. Although understanding the author may be due to the simple habit, it could keep consistent with the full manuscript including tables to avoid unnecessary conversion, comparison and misunderstanding for readers.

Author response 3: We thank you for pointing this out. We have modified all decimal places to three.

4. Line 261 to 264 was more suitable for discussion than conclusion. After all, there is no data on hospitalized children in this article, and no comparison to support this conclusion.

Author response 4: We thank you for your suggestion. We have changed this statement to discussion (Lines 248–252).

5. The interrupted time series analysis appears on Line 177, but its abbreviation ITSA first appeared on Line 87.

Author response 5: We thank you for pointing this out. We have modified the omitted text sections (Lines 88 and 178).
